# Tumor ABCC4-mediated release of PGE2 induces CD8^+^ T cell dysfunction and impairs PD-1 blockade in prostate cancer

**DOI:** 10.7150/ijbs.99716

**Published:** 2024-08-19

**Authors:** Le Li, Zheng Chao, Hao Peng, Zhiquan Hu, Zhihua Wang, Xing Zeng

**Affiliations:** 1 Department of Urology, Tongji Hospital, Tongji Medical College, Huazhong University of Science and Technology, Wuhan 430030, China.; 2 Institute of Organ Transplantation, Tongji Hospital, Tongji Medical College, Huazhong University of Science and Technology, Key Laboratory of Organ Transplantation, Ministry of Education, NHC Key Laboratory of Organ Transplantation, Key Laboratory of Organ Transplantation, Chinese Academy of Medical Sciences, Wuhan, 430030, China.

**Keywords:** ABCC4, pge2, prostate cancer, immune evasion, pd-1 blockade

## Abstract

Prostate cancer presents as an immunologically "cold" malignancy, characterized by a lack of response to immunotherapy in the majority of patients. The dysfunction of prostate tumor metabolism is recognized as a critical factor in immune evasion, resulting in reduced effectiveness of immunotherapeutic interventions. Despite this awareness, the precise molecular mechanisms underpinning metabolic dysregulation in prostate cancer and its intricate relationship with immune evasion remain incompletely elucidated. In this study, we introduce the multi-drug resistance protein ABCC4/MRP4 as a key player prominently expressed in prostate cancer, exerting a pivotal role in suppressing the activity of intratumoral CD8^+^ T cells. Depletion of ABCC4 in prostate cancer cells halts the release of prostaglandin E2 (PGE2), a molecule that diminishes the population of CD8^+^ T cells and curtails their cytotoxic capabilities. Conversely, constraining the activation of PGE2 signaling in CD8^+^ T cells effectively improved the efficacy of prostate cancer treatment with PD-1 blockade. During this process, downregulation of the JAK1-STAT3 pathway and depolarization of mitochondria emerge as crucial factors contributing to T cell anergy. Collectively, our research identifies the ABCC4-PGE2 axis as a promising target for reversing dysfunction within tumor-infiltrating lymphocytes (TILs) and augmenting the suboptimal responsiveness to immunotherapy in prostate cancer.

## Introduction

The incidence of prostate cancer ranks second among male malignancies globally, with over 375,000 deaths occurring each year^1^. Metastatic castration-resistant prostate cancer currently lacks effective treatment options, with patients facing a meager average survival period of merely 18 months^2^. In recent times, immune therapy, epitomized by immune checkpoint inhibitors, has displayed promising outcomes in combatting various solid tumors, instilling fresh optimism among patients^3^-^5^. Despite these advancements, prostate cancer remains categorized as an "immune cold" tumor, characterized by a diminished immune response rate and suboptimal therapeutic outcomes^6^-^8^. Consequently, there exists an imperative demand for pioneering breakthroughs in clinical practice to surmount this formidable challenge.

The anti-tumor immune response in prostate cancer predominantly relies on CD8^+^ T cells^9^. However, the presence of CD8^+^ T cells within the prostate cancer microenvironment is limited, and a significant proportion of these cells remain exhausted^10^. Therefore, the primary challenge in prostate cancer immunotherapy lies in elucidating the fundamental mechanisms leading to the anergy of these T cells. Recent studies have uncovered disparities in the regulation of oxidative phosphorylation and glycolysis within the tumor microenvironment, potentially stemming from the aberrant expression of metabolic-related proteins within prostate cancer cells, resulting in metabolic perturbations within the microenvironment^11^-^13^. Emerging research has highlighted the abnormal expression of numerous proteins within the ABCC family as a significant factor contributing to prostate cancer resistance against chemotherapy and androgen receptor (AR) inhibitors^14^. Among these proteins, ABCC4/MRP4 has been identified as a key player; however, the association between its expression and immune response in prostate cancer remains elusive.

ABCC4 belongs to the ATP-binding cassette (ABC) family of proteins, which can actively transport endogenous metabolites from cells, such as reduced glutathione, bile acids, steroid conjugates, and prostaglandin E2 (pge2)^15^. Pge2, synthesized extensively by cells through the action of Ptgs1/2, is released extracellularly and binds to Ptger2/4 receptors on the surface of neighboring cells, thereby initiating a cascade of downstream signaling events, one of which is the modulation of neuropathic pain^16^. Historically, the pervasive distribution of PGE2 led to the assumption that its functions were non-specific to organs or tissues. However, contemporary research indicates that prostaglandins possess a relatively brief half-life and are efficiently degraded by cells within the microenvironment upon secretion, hinting at the potential significance of examining the local effects of these small metabolites^16^. This is particularly pertinent in the context of the tumor microenvironment.

The latest research has unveiled the inhibitory effects of prostaglandin E2 (PGE2) on the local proliferation of CD8^+^ T cells within the context of colorectal cancer infiltration^17^. It has been demonstrated that blocking the production of PGE2 by targeting PTGS1/2 effectively restores the anti-cancer activity of CD8^+^ T cells^17^, ^18^. Nevertheless, the primary cellular source of PGE2 within the tumor microenvironment of prostate cancer remains to be definitively elucidated. Furthermore, the fundamental determinants governing the concentration of PGE2 within the tumor microenvironment, as well as the key mechanisms underpinning the disengagement of CD8^+^ T cells mediated by the PGE2-PTGER2 axis, continue to elude complete comprehension.

In this study, we aim to dissect the underlying mechanisms of the ABCC4-PGE2 axis in orchestrating the functional impairment of infiltrating CD8^+^ T cells in prostate cancer, while concurrently unraveling the metabolic perturbations characteristic of prostate cancer that contribute to immune suppression. We hope to furnish novel prognostic insights and therapeutic targets for the advancement of immunotherapeutic strategies tailored to prostate cancer.

## Materials and methods

### Patients and samples

We procured human prostate tissues, adjacent normal tissues, and bodily fluids in accordance with the ethical approvals granted by the Clinical Trial Ethics Committee of Tongji Hospital (Wuhan). Written informed consent was meticulously obtained from each patient involved in the study. Our cohort from Tongji Hospital comprised 289 patients with prostate cancer, for whom comprehensive follow-up data were available. The gene expression datasets for prostate adenocarcinoma (PRAD) were sourced from the cBioPortal for Cancer Genomics, utilizing the Gepia and Timer2.0 tools, for the analysis of The Cancer Genome Atlas (TCGA) cohort, ensuring the integration of robust and relevant genetic information into our study^19^, ^20^.

### Cell lines and cell culture

The human prostate cancer cell lines DU145, PC3, C4-2B, LNCap, and 22RV1 were procured from the American Type Culture Collection (ATCC), whereas the murine prostate cancer cell line RM1 was sourced from Procell Life Science & Technology (catalog number CL-0198). In order to ascertain the integrity and authenticity of the cell lines, we subjected them to rigorous testing for Mycoplasma contamination and cross-contamination between species, and their identities were verified through isoenzyme and short-tandem repeat analyses at the Cell Resource Center of Wuhan University, preceding the initiation of our study. The cells—DU145, PC3, C4-2B, LNCap, 22RV1, and RM1—were cultivated in RPMI-1640 medium (Gibco), supplemented with 100 IU/mL of Penicillin, 100 mg/mL of Streptomycin, 2 mM Glutamax, and 5% heat-inactivated fetal calf serum (HI-FCS). Lymphocytes from murine spleens and lymph nodes were isolated, and CD8^+^ T cells were subsequently purified by magnetic separation utilizing anti-APC magnet beads (Miltenyi Biotec) in conjunction with an anti-APC CD8 antibody (Biolegend). These isolated CD8^+^ T cells were then resuspended at a concentration of 1.0×10^6^ cells/mL and stimulated with monoclonal antibodies targeting CD3 and CD28 for a period of 48 hours. To isolate CD45^+^ T cells and tumor cells from mice, mononuclear cells were initially enriched from the tumors by density gradient centrifugation using Percoll (Biosharp). Following this, erythrocytes were lysed with a Cell Lysis Solution (Biosharp).

### Xenograft tumor model

The Institutional Animal Care and Use Committee at Tongji Hospital sanctioned all protocols pertaining to the mice. Male C57BL/6 and RagI mice, 6-8 weeks of age, were sourced from Cyagen Corporation and maintained in a pathogen-free environment at Tongji Hospital's Animal Facilities. To elicit tumor formation, 1.0×10^6^ RM1 cells were subcutaneously injected into the right flank of these mice. Tumor dimensions were routinely measured with calipers, and at designated intervals, tumors were harvested, weighed, and assessed for immune profiles by flow cytometry. To induce the depletion of CD8^+^ T cells, the murine subjects were administered with an anti-CD8 antibody, specifically the clone 2.43 (BE0061, BioXcell). As a comparator, the control group received 200 μg of rat IgG2b isotype control (BE0090, BioXcell). The antibodies were delivered via intraperitoneal injection every three days, commencing one day before the inoculation with tumor cells. For anti-PD1 immunotherapy, mice were inoculated with RM1 cells (wildtype or ABCC4-/-) on day 0 and received treatments of either 200ug of control IgG [rat IgG2a; BE0089 (BioXcell)] or anti-CD279 (PD1) antibody [BEIGENE, Beijing] via i.p. injection, administered 3-5 times at 3-day intervals beginning from the specified time point. The survival rate of the animals was determined by recording the percentage of mice that survived, with those having tumors larger than 2000 mm^3^ being classified as "dead." More detailed information are consistent with our previous work^21^.

### Flow cytometry

To facilitate the flow cytometric analysis of murine tissue specimens, the tumors were meticulously minced within Dulbecco's Modified Eagle Medium (DMEM) and subsequently subjected to enzymatic digestion with 2 milligrams of collagenase (Sigma, USA) for a duration of one hour at a temperature of 37°C. Following digestion, the cells were separated from tissue debris by passage through a 70-micron nylon strainer and then gently resuspended in a red blood cell lysis buffer (Biosharp) for a period of 3 minutes at ambient temperature. Post-lysis, the cells were carefully pelleted and resuspended in Phosphate-Buffered Saline (PBS) supplemented with 2% Bovine Serum Albumin (BSA), prior to being incubated with the pertinent antibodies for staining. Apoptosis flow cytometry was conducted using Annexin V-APC/7-AAD Apoptosis Kit (Multi Sciences, AP105).

### Western blot

The protein lysates were first diluted in Laemmli buffer and subjected to sonication, consisting of 15 cycles of 30-second intervals with pauses in between to prevent overheating. The samples were then heated to boiling and prepared for western blotting by the addition of β-mercaptoethanol and bromophenol blue. The protein samples were loaded onto the gel and electrophoresed before being transferred to membranes. These membranes were subsequently blocked using EpiZyme fast-blocking buffer and incubated overnight at 4°C with specific primary antibodies within the blocking buffer, which was supplemented with 0.2% Tween-20. The primary antibodies utilized included those targeting ABCC4 (Catalog Number: A2198, ABCLONAL, China), β-Actin (Catalog Number: AC004, ABCLONAL, China), Stat3 (Catalog Number: A16975, ABCLONAL, China), p-Stat3 (Catalog Number: AP0474, ABCLONAL, China), Jak1 (Catalog Number: A5534, ABCLONAL, China), p-Jak1(Catalog Number: ab138005, ABCAM, USA), Stat5 (Catalog Number: 381427, ZENBIO, China) and p-Stat5 (Catalog Number: 381125, ZENBIO, China). Further details regarding the antibodies and their usage are provided within the Supplementary materials and methods section. For the secondary antibody incubation phase, the membranes were either treated with a fast blocking buffer at ambient temperature for a duration of 10 to 15 minutes or directly immersed in a diluent-blocking buffer, which was compounded with 0.2% Tween-20 and 0.01% SDS, for a period of 1 hour at room temperature. Following this blocking procedure, the membranes were exposed to the secondary antibodies under optimized conditions to ensure specific and sensitive detection. Subsequent to the secondary antibody incubation, the membranes were imaged utilizing a fluorescence detection system on a Biorad Imager, which provided high-resolution visualization of the protein bands of interest. The captured images were then processed and refined using Adobe Photoshop CC 2023 software to enhance clarity and facilitate detailed analysis and presentation.

### Immunohistochemistry

In the meticulous preparation of tissue microarrays for analytical examination, the initial step involved deparaffinization through baking the specimens at a temperature of 60 °C for a duration of 30 minutes. This was succeeded by a successive series of washes with Citrus Clearing Solvent, followed by progressively decreasing concentrations of ethanol—100%, 95%, and 70%—to ensure thorough removal of wax and contaminants. To facilitate antigen retrieval, the slides were subjected to intense heat treatment within a pressure cooker, immersed in a pH 6.0 citrate solution (Vector Laboratories), and exposed to temperatures of 125 °C for 30 seconds, followed by a rapid cooling to 90 °C for an additional 10 seconds. Post-retrieval, the slides underwent permeabilization with 0.1% Tris Buffered Saline with Tween-20 (TBS-T) for 5 minutes, and were then blocked to minimize non-specific binding, first with Bloxall peroxidase block for 10 minutes and subsequently with horse serum a 20-minute interval. The tissue arrays were then incubated overnight with the primary antibody, ensuring ample time for antibody-antigen interaction. This was followed by a 30-minute incubation with the secondary antibody at room temperature to amplify the signal. The Vectastain Elite ABC reagent was subsequently applied for a duration of 30 minutes, and the slides were stained with a 1:1 mixture of ImPACT DAB EqV reagent 1 (Chromogen) and reagent 2 (Diluent). After washing the slides with water three times, we applied the bluing reagent once to achieve a clear contrast for visual assessment. The dehydration process was then meticulously undertaken, with the tissue sections subjected to a graduated series of ethanol washes to progressively remove water and prepare the slides for mounting. This began with five iterations in 70% ethanol, followed by ten cycles in 95% ethanol, and concluded with two, 10-minute immersions in 100% ethanol to ensure complete dehydration. Ultimately, the slides were securely mounted using Cytoseal 60 (Biosharp) to preserve the tissue sections and prevent degradation. The mounted slides were then imaged using a BioTek Cytation 5 imaging system, capturing high-resolution visuals for detailed analysis and documentation.

More detailed materials and methods can be found in the Supplementary Materials.

### Statistical analysis

The data were meticulously quantified using the GraphPad Prism software suite. To ascertain statistical significance between the two comparator cohorts, a two-tailed, unpaired Student's t-test was administered. For the purpose of comparing outcomes across multiple groups, each harboring a single experimental variable, a one-way Analysis of Variance (ANOVA) was resorted to. In experimental designs encompassing two distinct parameters, a two-way ANOVA was executed to discern statistically significant differences among the groups. The survival curves were subjected to statistical scrutiny by means of the log-rank test, commonly referred to as the Mantel-Cox test. A p-value threshold of less than 0.05 was established to denote statistical significance of the observed results.

## Results

### ABCC4 depletion in prostate cancer inhibits tumor growth

**Figure 1:**
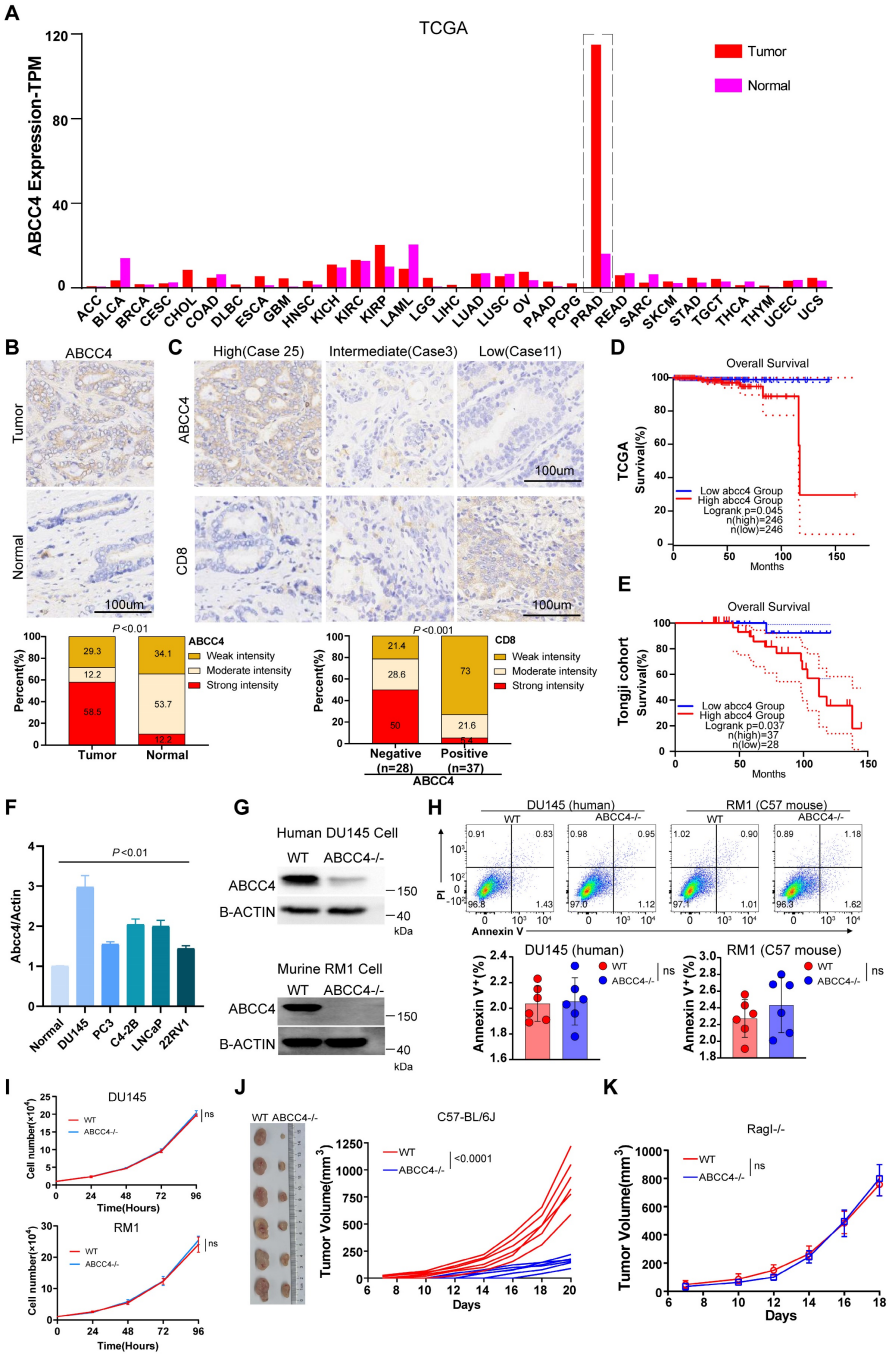
**ABCC4 depletion in prostate cancer inhibits tumor growth.** (A) ABCC4 expression levels across varieties of cancer based on TCGA database. (B) Top: representative IHC results of ABCC4 expression in samples of prostate cancer and adjacent normal tissues; Bottom: statistical analysis of ABCC4 expression in samples of prostate cancer (n=41) and adjacent normal tissues (n=41) from Tongji Cohort. (C) Top: representative IHC results of ABCC4 expression and CD8 expression in samples of prostate cancer from Tongji Cohort; Bottom: statistical analysis of CD8 expression in samples of prostate cancer with negative/low ABCC4 expression (n=28) and positive/high ABCC4 expression (n=37) from Tongji Cohort. (D) and (E) Overall survival of PRAD patients with high/low expression of ABCC4 based on (D)TCGA database and (E)Tongji Cohort. (F) QPCR results showing ABCC4 expression in normal prostate tissues and several human prostate cancer cell lines including DU145, PC3, C4-2B (hormone-insensitive) and LNCap, 22RV1(hormone-sensitive). (G) Western blot showing results of ABCC4 wildtype and knock out in DU145 cells (human) and RM1 cells (mouse) of prostate cancer. (H) Flowcytometry results of ABCC4 wildtype and knock out in DU145 cells and RM1 cells. (I) CCK8 results of ABCC4 wildtype and knock out in DU145 cells and RM1 cells. (J) Tumor growth of RM1 cell transplanted subcutaneously in C57/BL6 mice. (K) Tumor growth of RM1 cell transplanted subcutaneously in Rag1 mice (Rag1-/-). Data in F-H are pooled from three independent experiments and depicted as the mean ± s.e.m. Plots in I and J show data for 1 tumour representative of n = 6 tumours from 2 independent experiments. P values are from two-way analysis of variance (ANOVA) with Bonferroni's correction for multiple testing (I,J,K) or unpaired t-tests t test (B,C,H). NS, not significant (P ≥ 0.05).

We first examined ABCC4 expression across all kinds of cancer in TCGA database and found that it was only in prostate cancer extremely high expressed compared to that in adjacent normal tissues (Figure 1A). We also confirmed the ABCC4 expression difference in tumors and adjacent normal tissues of prostate cancer (Figure 1B). Moreover, high expression of ABCC4 in prostate cancer cells was assosiated with low CD8^+^ T cell infiltration (Figure 1C). Next, we analyzed patients' survival and found that low expression of ABCC4 indicated better overall survial for prostate cancer patients both in TCGA database and in our own Tongji Cohort (Figure 1D and 1E). We also examined the ABCC4 expression in multiple human prostate cancer cell lines and identified DU145 with highest ABCC4 expression (Figure 1F). To investigate the specific function of ABCC4, we depleted it in both DU145 cells (human prostate cancer) and RM1 cells (murine prostate cancer) (Figure 1G). However, genetic ablation of ABCC4 did not promote apoptosis (Figure 1H) nor inhibit growth (Figure 1I) of prostate cancer cells *in vitro*. Next, we transplanted RM1 cells (ABCC4-/-) into C57 mice with complete immune system and observed significant growth restriction compared to control group (Figure 1J), while observed similar tumor growth rates in Rag-I mice with incomplete immune systems (Figure 1K). This suggests that ABCC4 ablation inhibits prostate cancer in an inmmune response-dependent manner.

### ABCC4 depletion in prostate cancer boosts anti-tuomr immunity of CD8^+^ T cells

**Figure 2:**
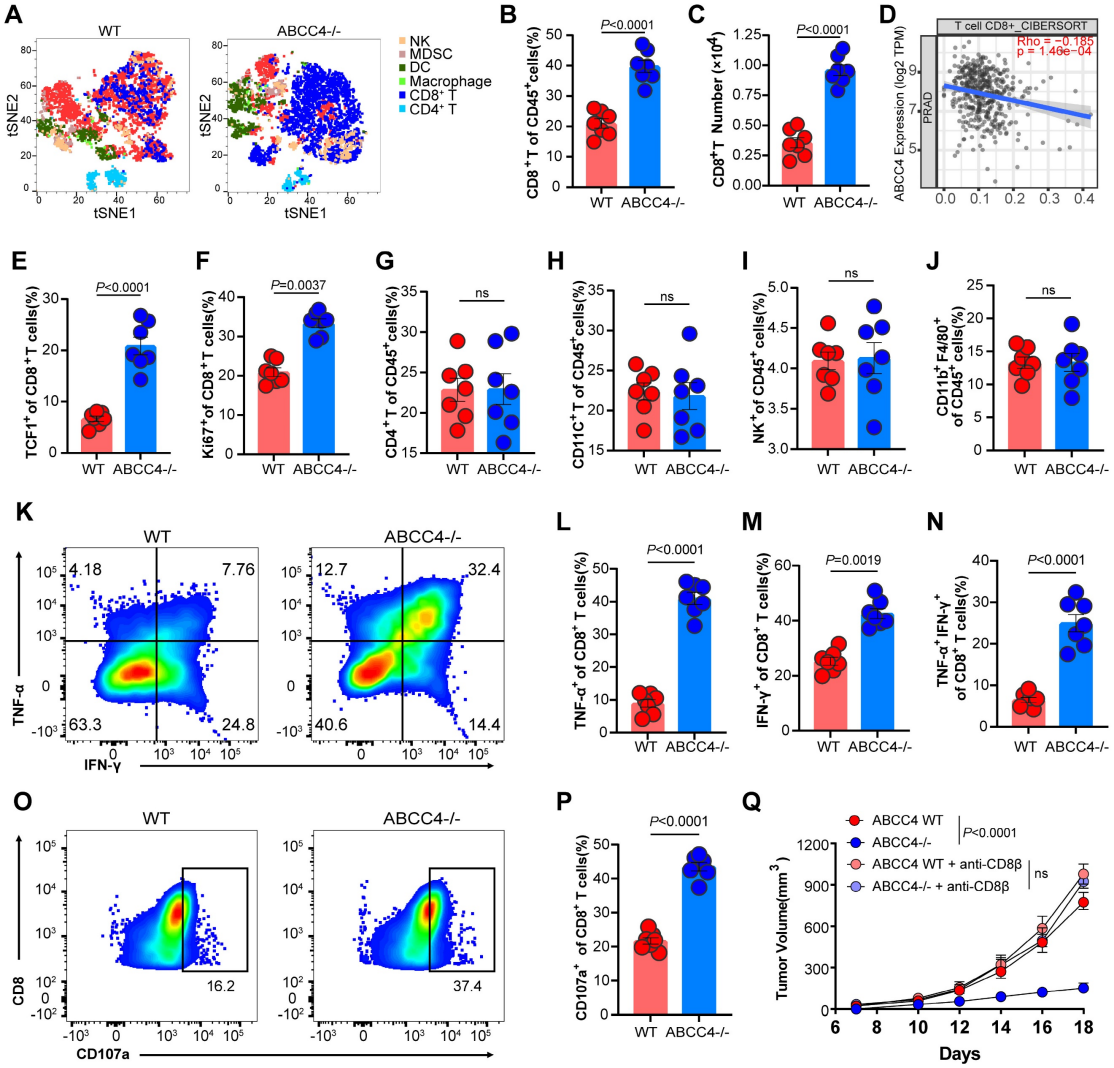
** ABCC4 depletion in prostate cancer boosts anti-tuomr immunity of CD8^+^ T cells.** (A) Tsne results of intratumoral subclusters of immune cells in murine prostate cancer transplanted with RM1 cells (ABCC4 wildtype and knock out). (B-C) Quantification of (B)percentages and (C) numbers of tumor-infiltrating CD8^+^ T cells in murine prostate cancer (n=7). (D) Pearson correlation of ABCC4 expression and CD8 score in PRAD based on CIBERSORT in Timer2.0. (E-F) Quantification of percentages of (E)TCF1^+^CD8^+^ and (F)Ki67^+^CD8^+^ cells in CD8^+^ T cells. (G-J) Quantification of percentages of (G) CD4^+^, (H) CD11C^+^, (I) NK^+^ and (J) CD11b^+^ F4/80^+^ out of CD45^+^ cells (n=7). (K) Representative flow plots of IFN-γ^+^ and TNF-α^+^ cells out of CD8^+^CD45^+^ cells. (L-N) Quantification of percentages of (L) TNF-α^+^, (M) IFN-γ^+^ and (N) TNF-α^+^IFN-γ^+^ out of CD45^+^ cells (n=7). (O-P) (O)Representative flow plots and (P)quantification of percentages of CD107a^+^CD8^+^ cells out of CD8^+^CD45^+^ cells (n=7). (Q) Tumor growth of RM1 cell transplanted subcutaneously in C57/BL6 mice (n=5). Error bar represents mean ± SEM. Statistical significance was determined by unpaired Student's t-test for (B,C,E-J,L-N,P) and two-way analysis of variance (ANOVA) with Bonferroni's correction for Q. NS, not significant (P ≥ 0.05).

To further investigate the specific effects of ABCC4 depletion in tumor cells on the tumor immune microenvironment, we used tSNE plots to indicate the landscape (Figure 2A). We found the proportions and absolute numbers of tumor-infiltrating CD8^+^ T cells were both elevated upon ABCC4 depletion in RM1 cells (Figure 2B-C). The cibersort results of tumor ABCC4 expression and CD8 scores were consistently negative correlated in PRAD (Figure 2D). Moreover, TCF1 expression representative of stemness (Figure 2E) and Ki67 representative of preliferative capacity (Figure 2F) of CD8^+^ T cells were both upregulated in the ABCC4-KO group. Meanwhile, other main subclusters of immune populations were not alterd (Figure 2G-J). Next, we found the expression of TNF-α, IFN-γ and CD107a representative of cytotoxicity in CD8^+^ T cells were all elevated (Figure 2K-P) in the the ABCC4-KO group, implying that genetic ablation of ABCC4 in tumor cells could restore the anti-tumor effects of CD8^+^ T cells. Furthermore, *in vivo* depletion of CD8^+^ T cells in C57 mice caused similar tumor growth rates in the ABCC4-KO group and control group (Figure 2Q), suggesting that the supressive effects of ABCC4 ablation in tumor cells relied on restoration of CD8^+^ T cells.

### ABCC4-mediated Pge2 release from tumor induces apoptosis and dysfunction of CD8^+^ T cells

**Figure 3:**
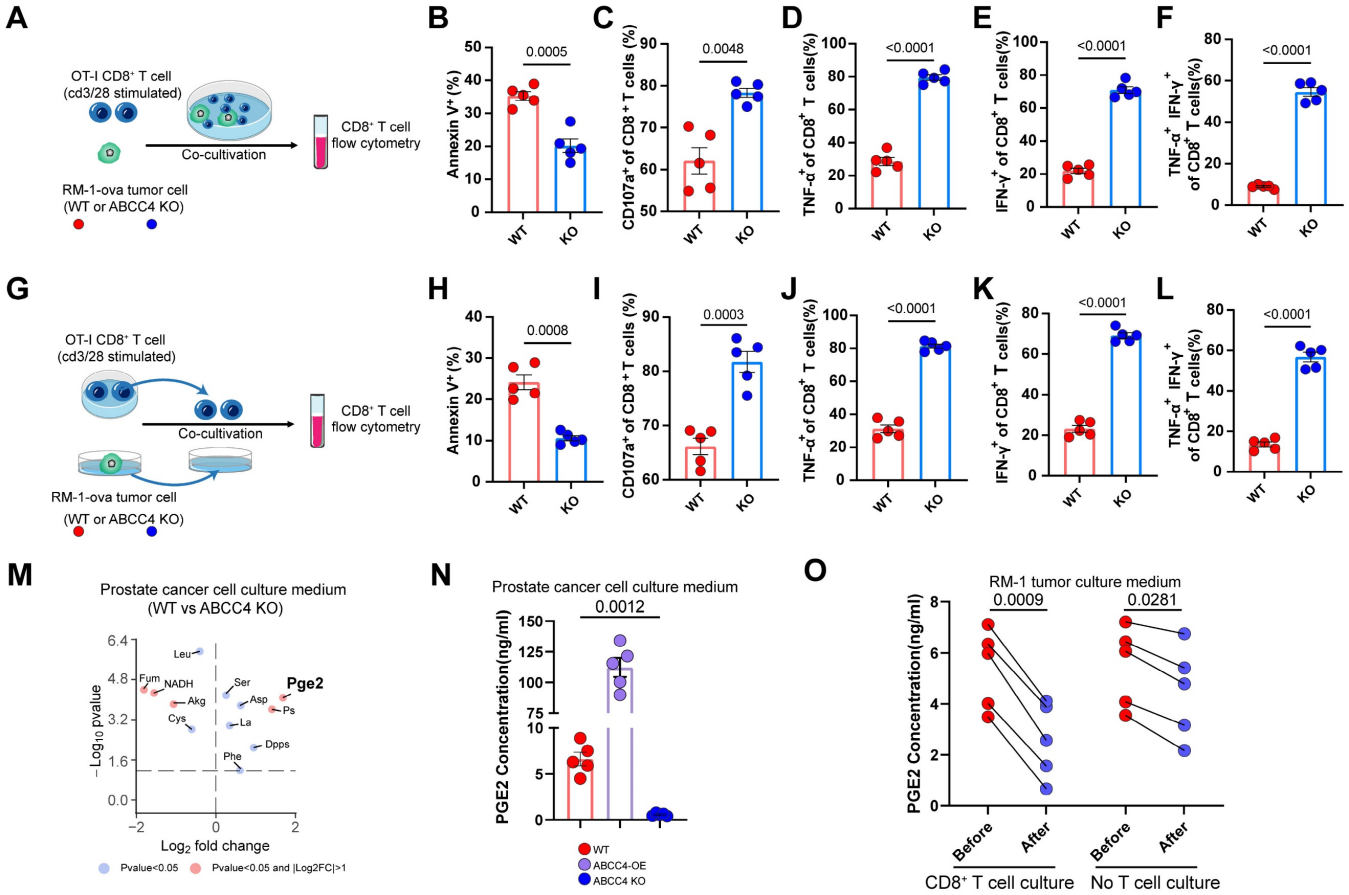
** ABCC4-mediated Pge2 release from tumor induces apoptosis and dysfunction of CD8^+^ T cells.** (A) Graphical presentation illustrating direct co-culture of CD8^+^ t cell and RM1 cell. (B-F) Quantification of (B) apoptosis and (C-F) cytotoxic function of CD8^+^ T cells co-cultured with RM1 cells (n=5). (G) Graphical presentation illustrating co-culture of CD8^+^ t cell and the culture medium of RM1 cell. (H-L) Quantification of (H) apoptosis and (I-L) cytotoxic function of CD8^+^ T cells co-cultured with culture medium of RM1 cells (n=5). (M) Differential analysis of metabolics identification of metabolites in culture medium of RM1 cells. (N) Pge2 concentration in RM1 cell culture medium. OE: overexpression (n=5). (O) Pge2 concentration in RM1 cell culture medium before and after CD8^+^ T cell/no T cell culture (n=5). Error bar represents mean ± SEM. Statistical significance was determined by unpaired Student's t-test for (B-F,H-L), paired Student's t-test for (O) and one-way analysis of variance (ANOVA) with Tukey's multiple-comparison test for (N). NS, not significant (P ≥ 0.05).

To learn more of the assosiation of ABCC4 expression in tumor cells and T cells, we directly co-cultured stimulated CD8^+^ T cells with RM1 cells (ABCC4-/- or WT) (Figure 3A). Consequently, we found that the apoptosis of CD8^+^ T cells were diminished (Figure 3B) and cytotoxic capacity were upregulated (Figure 3C-F). Next, we co-cultured CD8^+^ T cells with the cell culture medium supernatants of RM1 cells (ABCC4-/- or WT) (Figure 3G). Intriguingly, similar function results were observed as those in the direct co-culture system (Figure 3H-L), and this implied that changes in the medium supernants after ABCC4 knockout restored CD8^+^ T cell function. Consequently, pge2 were identified significantly reduced in the culture medium of ABCC4 knockout RM1 cells (Figure 3M). We verified the influence of ABCC4 overexpression and knock-out on pge2 concentration in culture medium (Figure 3N). Furthermore, we also examined the concentration changes of pge2 in the RM1 medium supernatants after CD8^+^ T cell culture and found it was degraded significantly within 60 minutes (Figure 3O). Considering the short half-life period of pge2, it is reasonable to speculate that only endless production and release of pge2 accumulated in a crowded place could induce disturbed function of CD8^+^ T cells.

### PGE2-EP2/EP4 axis limits CD8^+^ T cell viability and function

**Figure 4:**
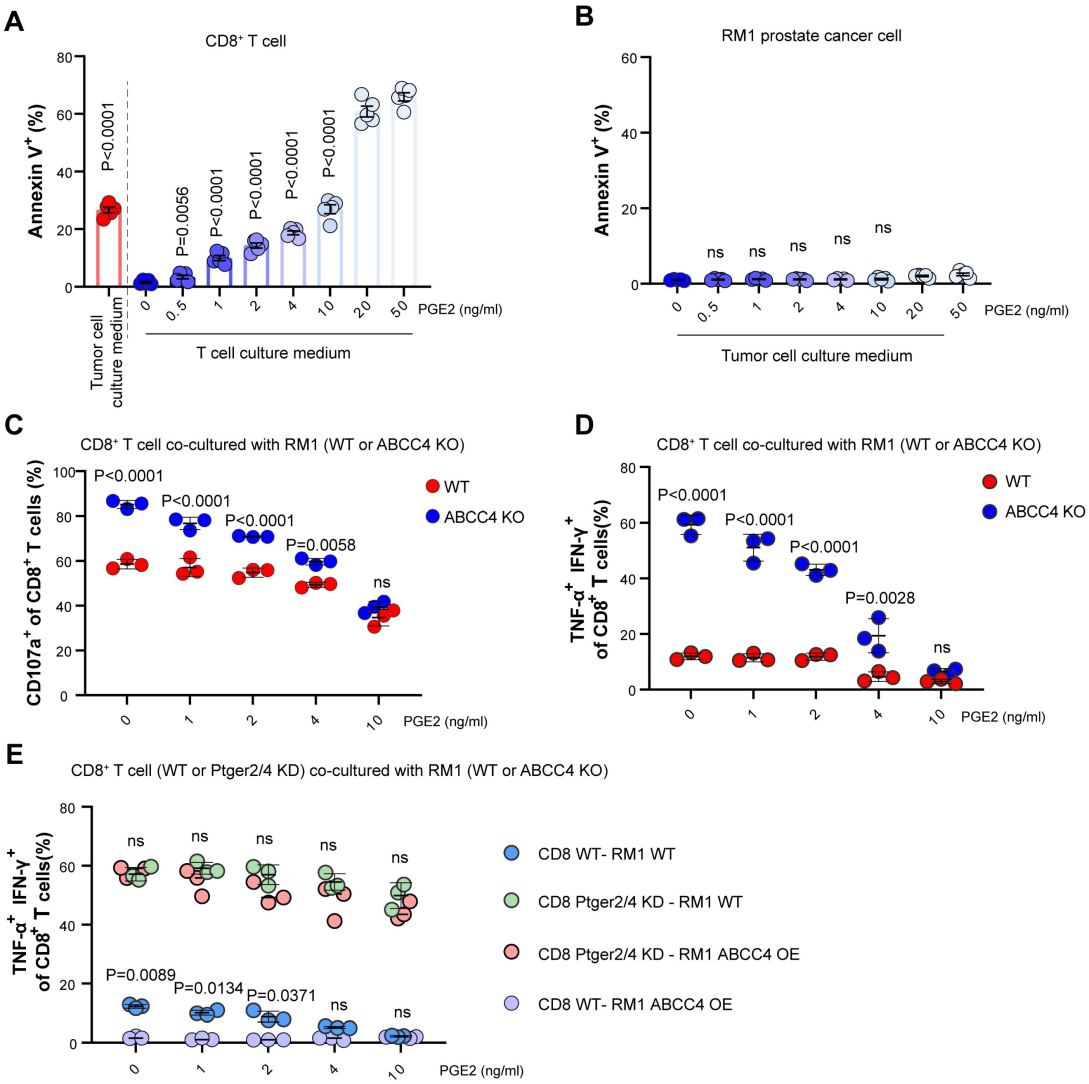
** PGE2-EP2/EP4 axis limits CD8^+^ T cell viability and function.** (A) Apoptosis of CD8^+^ T cells cultured with tumor culture medium or pge2 of different concentration (n=5). (B) Apoptosis of RM1 cells cultured with pge2 of different concentration (n=5). (C) CD107a expression of CD8^+^ T cells co-cultured with RM1 cells in medium with pge2 of different concentration (n=3). (D-E) Dual expression of IFN-γ and TNF-α in CD8^+^ T cells co-cultured with RM1 cells in medium with pge2 of different concentration (n=3). KD: knockdown. Error bar represents mean ± SEM. Statistical significance was determined by one-way analysis of variance (ANOVA) with Tukey's multiple-comparison test for (A-B), unpaired Student's t-test for (C-E). NS, not significant (P ≥ 0.05).

To validate the inbitory effects of pge2 on CD8^+^ T cells, we added pge2 of different concentration to CD8^+^ T cells and found the apoptosis was increasingly induced as the concentration rised (Figure 4A), but did not alter RM1 cell viability (Figure 4B). Moreover, supplementation of pge2 to the co-culture system of CD8^+^ T cells and RM1 cells (WT or ABCC4 KO) eliminated the boosting effects of ABCC4 depletion in tumor cells on CD8^+^ T cells (Figure 4C and 4D), strongly supporting the speculation that the effects of ABCC4 on anti-tumor immunity depended on its regulation of pge2 release from prostate cancer cells. Next, we knocked down the Ptger2 and Ptger4 in CD8^+^ T cells (encoding EP2/4 receiving pge2 signaling) and found supplementation of pge2 no longer inhibited CD8^+^ T cell function (Figure 4E), underlying the determining roles of ABCC4-PGE2-EP2/EP4 axis in regulating anti-tumor functions of CD8^+^ T cells in prostate cancer.

### Activation of PGE2-EP2/EP4 axis induces glucose metabolism reprogramming and mitochondrial depolarization

**Figure 5:**
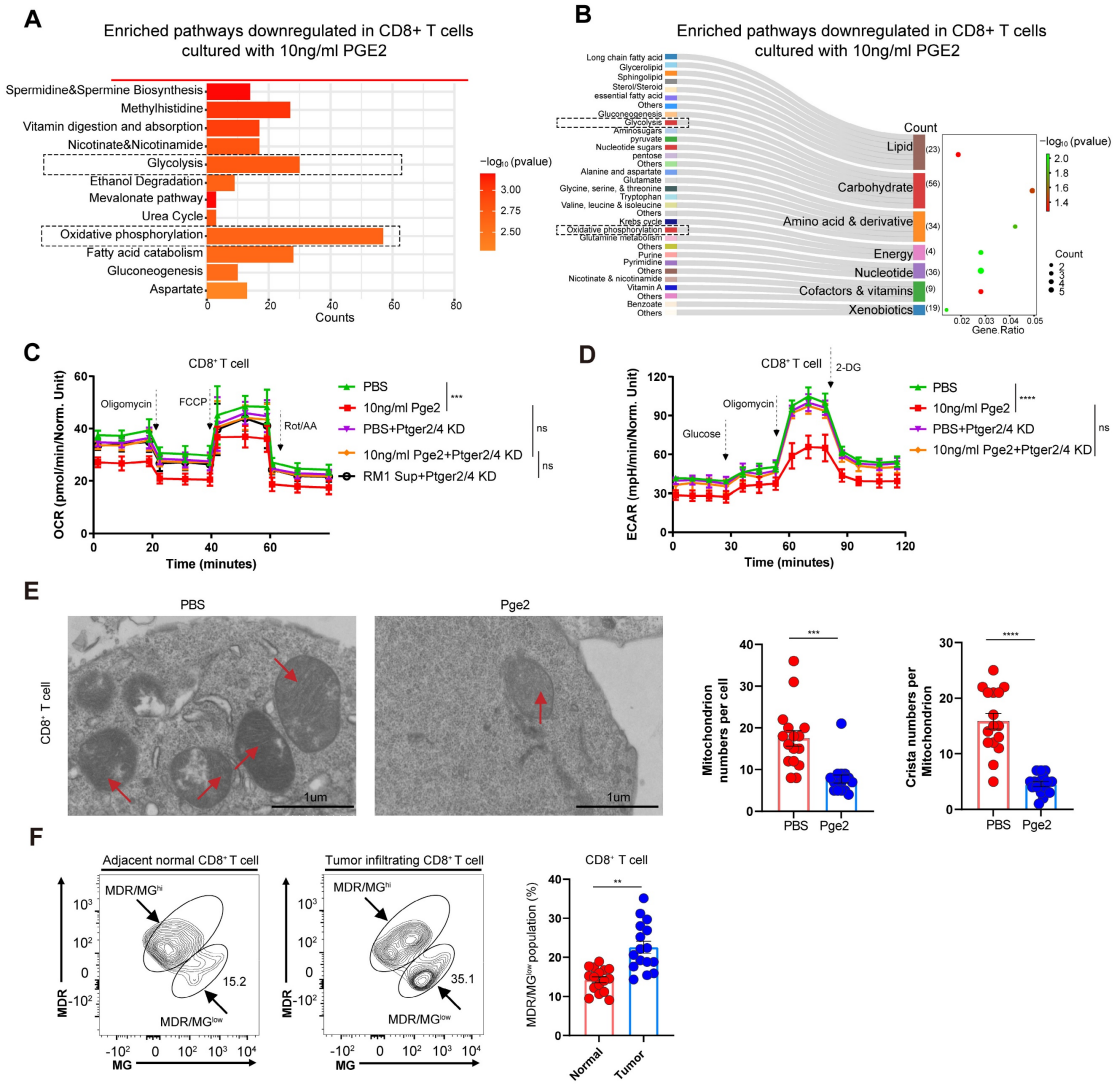
** Activation of PGE2-EP2/EP4 axis induces glucose metabolism reprogramming and mitochondrial depolarization.** (A-B) KEGG pathway downregulated in CD8^+^ T cells cultured with 10ng/ml pge2 versus 0ng/ml. (C) Oxygen Consumption Rate (OCR) of CD8^+^ T cells with different culture condition (n=4). Sup: supernatant. (D) Extra Cellular Acidification Rate (ECAR) of CD8^+^ T cells with different culture condition (n=4). (E) Left: representative transmission electron microscopy of CD8^+^ T cells treated with PBS or 10ng/ml pge2 (n=15). Right: quantification of mitochondrion numbers and crista numbers per mitochondrion of CD8^+^ T cells treated with PBS or 10ng/ml pge2. (F) Left: representative flow spots of CD8^+^ T cells stained with MitoTracker Green(MG) and MitoTracker Deep Red(MDR) (n=15). Right: quantification of MDR/MGlow populations of CD8^+^ T cells in tumor and adjacent normal tissues. Error bar represents mean ± SEM. Statistical significance was determined by unpaired Student's t-test. NS, not significant (P ≥ 0.05). **P < 0.001, and ****P < 0.0001

To study the metabolic changes of CD8^+^ T cells after pge2 supplementation, KEGG analysis was conducted (Figure 5A and 5B). To verify the effects of the PGE2-EP2/EP4 axis on glucose metabolism, seahorse was conducted and we found retardation of the PGE2-EP2/EP4 axis restored the glycolysis and oxidative phosphorylation of CD8^+^ T cells (Figure 5C and 5D). Moreover, this reprogrammed metabolism could result from reduced mitochondrial numbers and crista numbers (Figure 5E). Besides, clinical samples also showed increased MDR/MG^low^ populations of CD8^+^ T cells in tumors compared to those in adjacent normal tissues (Figure 5F), indicating that the dysregulated function of CD8^+^ T cells in prostate cancer could be triggered by depolarized mitochondrion.

### PGE2 signaling inhibits JAK1-STAT3 pathway in CD8^+^ T cells

**Figure 6:**
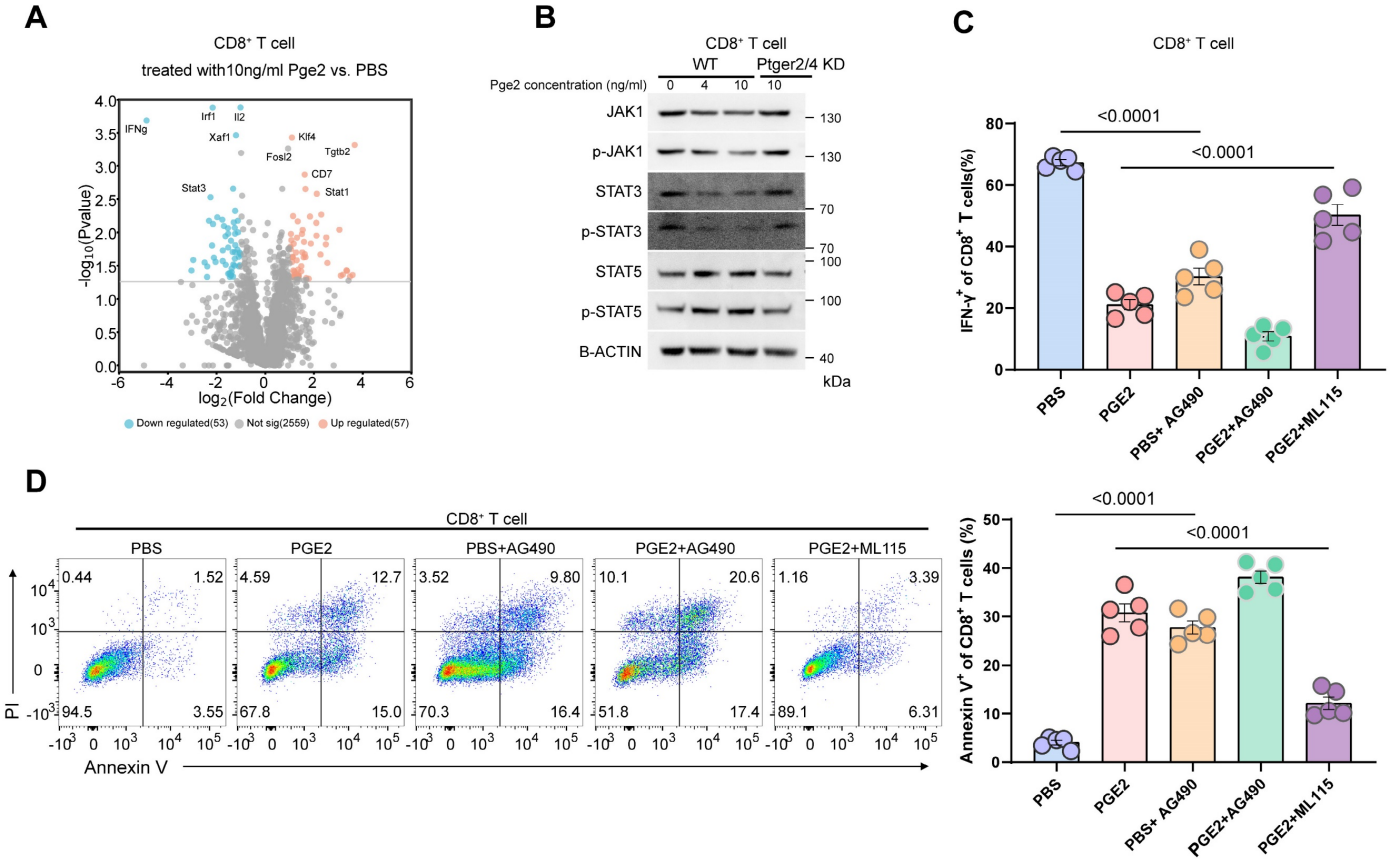
** PGE2 signaling inhibits JAK1-STAT3 pathway in CD8^+^ T cells.** (A) Volcano plots indicating up/down-regulated genes in CD8^+^ T cells after treatment with 10ng/ml pge2. (B) Western blot results of CD8^+^ T cells. (C) Quantification of IFN-γ expression of CD8^+^ T cells (n=5). AG490: STAT3 inhibitor; ML115: STAT3 activator. (D) Left: representative flow plots of CD8^+^ T cells treated with pbs, 10ng/ml pge2 and/or other small molecule drugs (n=5). Right: quantification of annexin-v^+^ CD8^+^ T cells treated as left. Error bar represents mean ± SEM. Statistical significance was determined by one-way analysis of variance (ANOVA) with Tukey's multiple-comparison test.

We conducted volcano analysis of differentially expressed genes of CD8^+^ T cells after pge2 treatment, and found that stat3 and ifng was downregulated (Figure 6A). Considering ifng was reported to be regulated by stat3 and stat5^22^, ^23^, we compared their expression in CD8^+^ T cells treated with different concentration of pge2 and found that only p-JAK1, STAT3 and pSTAT3 were downregulated instead of stat5 (Figure 6B). Targeted inhibition of STAT3 signaling with AG490 aggravated the anergy and apoptosis of CD8^+^ T cells while stimulation of STAT3 with ML115 restored CD8^+^ T cells function and viability to some extent (Figure 6C and 6D).

### ABCC4 depletion enhanced PD-1 blockade and prolonged survival in mice with prostate cancer

**Figure 7:**
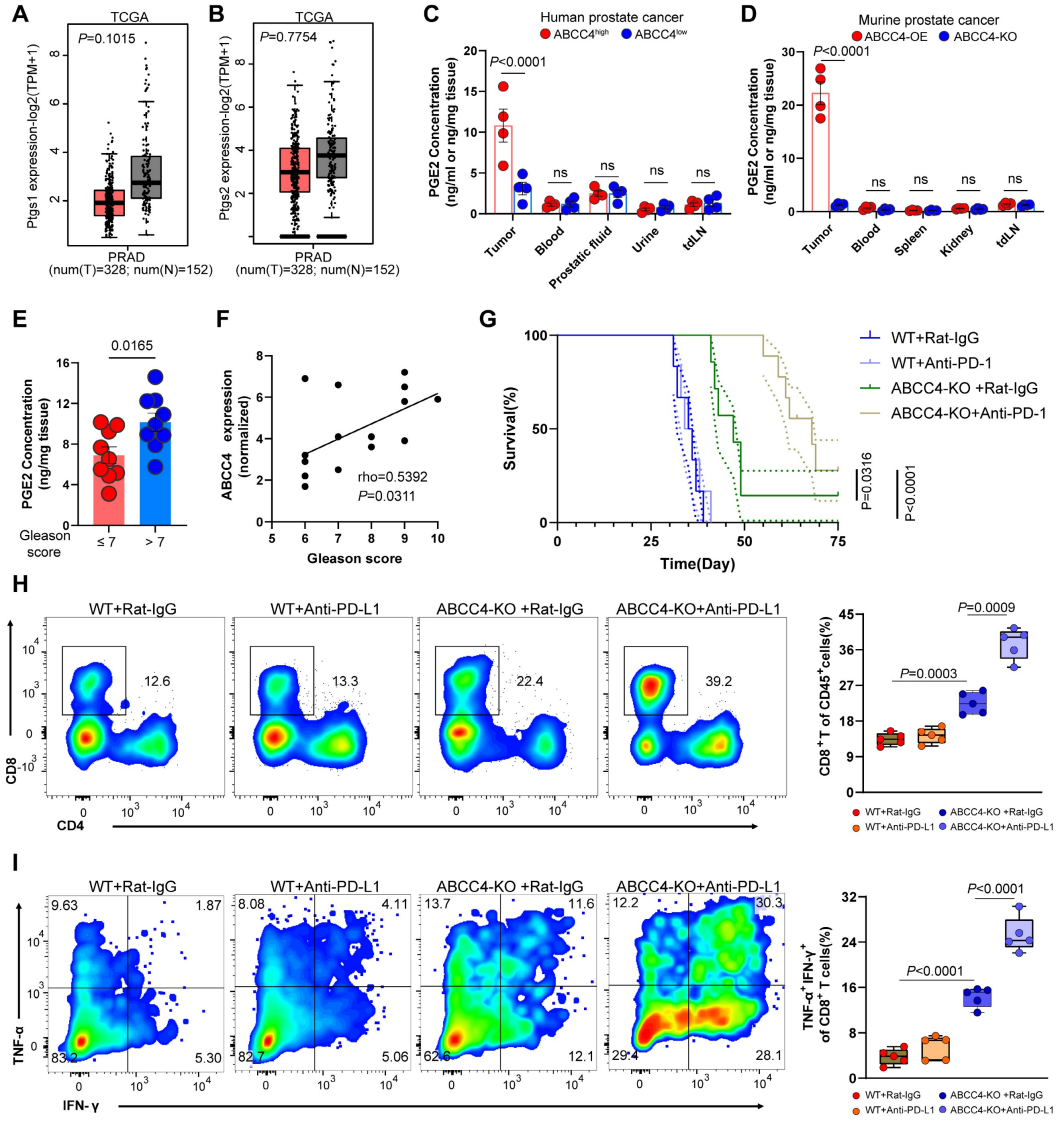
** ABCC4 depletion enhanced PD-1 blockade and prolonged survival in mice with prostate cancer.** (A-B) Ptgs1/2 expression in tumors and normal tissues in PRAD based on TCGA database from gepia. (C-D) Pge2 concentration in tumors and other organs or body fluid in (C) human prostate cancer or (D) murine prostate cancer (n=4). (E) Pge2 concentration in human prostate tumors grouped according to gleason score of 18 patients. (F) Pearson correlation between ABCC4 expression and gleason score of prostate cancer in 15 patients. (G) Overall survival of C57 mice transplanted with prostate cancer (n=7-8). (H) Representative flow plots (left) and quantification (right) of tumor infiltrating CD8^+^ T cell populations in murine prostate cancer (n=5). (I) Representative flow plots (left) and quantification (right) of TNF-α/IFN-γ expression of tumor infiltrating CD8^+^ T cell populations in murine prostate cancer (n=5). Error bar represents mean ± SEM. Statistical significance was determined by unpaired Student's t-test for (A-E), log-rank (Mantel-Cox) test for (G), and one-way analysis of variance (ANOVA) with Tukey's multiple-comparison test for(H-I).

**Figure 8:**
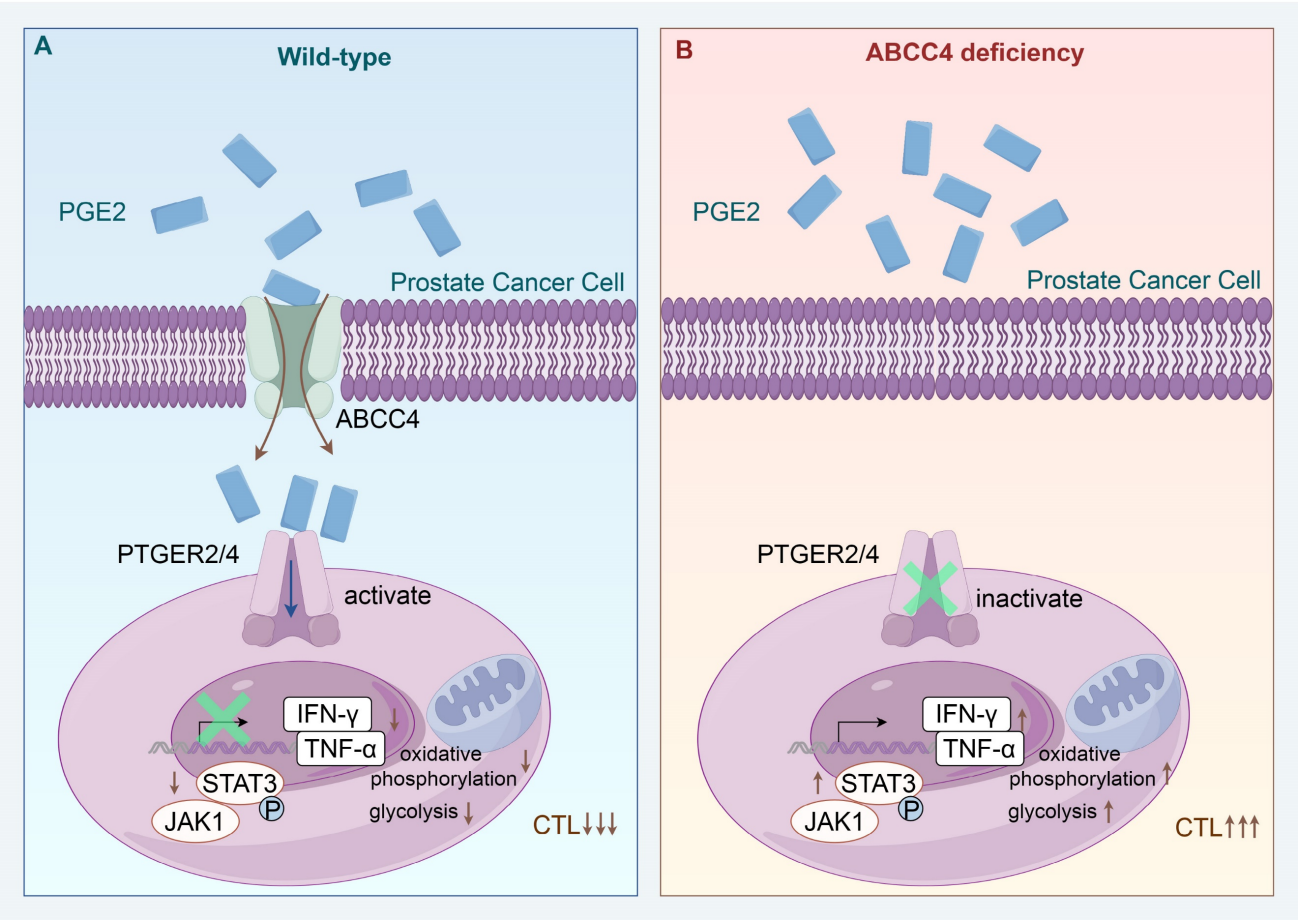
Schematic model depicting that ABCC4 deficiency in prostate cancer restores anti-tumor cytotoxicity of CD8^+^ T cells.

Since the expression of ptgs1/2 producing pge2 showed no difference in tumors and adjacent normal tissues based on TCGA database (Figure 7A and 7B), it is reasonable to owe the enrichment of pge2 in the tumor microenvironment to high expression of ABCC4. Comparement of pge2 concentration in other various organs and body fluids from both human and mice with prostate cancer did not show difference compared to those from healthy cases (Figure 7C and 7D), further supporting that ABCC4 guarded the pge2 release and focal concentration in tumors. We also found that higher intratumoral pge2 concentration and ABCC4 expression was assosiated with higher gleason scores (Figure 7E and 7F), suggesting the ABCC4-PGE2 could be used as specific diagnostic and predictive markers of prostate cancer and tumor grades. Moreover, ABCC4 ablation in tumors significantly enhanced PD-1 blockade efficacy and greatly improved survival of prostate cancer mice (Figure 7G), and also dramatically restored anti-tumor immunity of CD8^+^ T cells (Figure 7H and 7I), indicating that the ABCC4-PGE2 axis could be promising targets to overcome immune evasion and resistance to immunotherapy in prostate cancer (Figure 8).

## Discussion

Prostate cancer, uniquely among solid tumors, exhibits a distinct association with androgens^24^. Consequently, androgen blockade has emerged as the standard therapeutic modality for the vast majority of prostate cancer patients^24^. Nonetheless, a significant challenge arises as many individuals with advanced stages of the disease inevitably develop resistance to this treatment, prompting a search for alternative therapeutic strategies^24^. Immunotherapies, typified by PD-1 inhibitors, have been adoptively integrated into the treatment arsenal for various solid tumors, owing to their selective targeting and profiles of minimal adverse effects^24^, ^25^. However, these interventions have not proven efficacious for the survival of the majority of prostate cancer patients^25^.

The mechanisms underlying PD-1 inhibitors revolve around the modulation of PD-1 signaling in CD8^+^ T cells during the intermediate and late stages of tumor progression, thereby reactivating their antitumor capabilities^26^. This restoration of function is exclusive to T cells with intact metabolic homeostasis, as T cells within prostate cancer microenvironments frequently exhibit dysregulated glucose metabolism, such as reduced glycolysis or oxidative phosphorylation, which impairs cellular energy metabolism^27^. Therefore, unraveling the critical upstream regulatory mechanisms that contribute to this metabolic chaos is important to ameliorating the suboptimal therapeutic outcomes associated with prostate cancer.

Our findings delineate that the pge2 signaling pathway plays a pivotal role in mediating the metabolic abnormalities observed in CD8^+^ T cells. Suppression of the EP2/EP4 receptor signaling effectively reverses the energy deficit experienced by T cells and restores their cytotoxic potential. The significance of the PGE2-EP2/EP4 axis has been corroborated by several recent studies, which underscore its indispensable role in tumor immunity^17^, ^18^, ^28^. A predominant challenge in these studies is the ubiquitous expression of EP2/EP4 in various immune cell subsets within the tumor microenvironment, posing a substantial challenge for the clinical application of targeted EP2/EP4 receptor inhibition due to the anticipated deleterious side effects on normal immune function.

Pge2, the metabolite that triggers the EP2/EP4 signaling cascade, is a ubiquitously synthesized compound in numerous cellular contexts within the human body^29^. Consequently, research focusing on the local tumor microenvironment has generated controversy, suggesting that the systemic effects of these widely distributed substances cannot be confined to the local tumor microenvironment. However, our investigation demonstrates that the levels of pge2 in the extracellular fluids of non-tumoral tissues and organs in both humans and mice are substantially lower than those in tumor microenvironments. This discrepancy may be attributed to the cancer cells' reported heightened synthesis^17^, ^18^, the enhanced transport capacity observed in our studies, or the shoer half-life of pge2^30^, which leads to its degradation during measurement. Regardless, our work, along with several prior studies^17^, ^18^, ^28^, supports the notion that molecules previously associated solely with pain signaling also serve as critical tools utilized by tumor cells to subvert immune surveillance.

The distinct aspect of our approach compared to prior research is the nature of our pge2 signaling inhibition strategy. Predominant prior efforts have focused on reducing pge2 levels within the tumor microenvironment through the inhibition of its synthesis^17^, ^18^, whereas we have achieved similar outcomes by intercepting its cellular export. It is noteworthy that the divergent strategies emanate from the dissimilarities inherent in various solid tumors. In prostate cancer, we observed that the expression of pge2 synthesis genes, ptgs1/2, is not elevated or is even diminished relative to normal tissues, suggesting that the heightened pge2 concentrations within the prostate cancer microenvironment do not arise from enhanced synthesis. Conversely, the marked overexpression of the transporter protein ABCC4 in prostate cancer, compared to other solid tumors, indicates that the preferential export of pge2 from cancer cells is the predominant source of its elevated levels in prostate cancer (over-export). Moreover, due to its high expression specificity, ABCC4 has great potential to become a target for prognosis prediction and intervention therapy of prostate cancer.

Our investigation has elucidated that the ABCC4-PGE2-EP2/EP4 signaling axis triggers downstream mitochondrial depolarization and glucose metabolic reprogramming, which serves as a crucial underlying mechanism for the functional impairment and immunosuppression of CD8^+^ T cells within the prostate cancer microenvironment. The relative stability of mitochondrial membrane potential is fundamental to maintaining mitochondrial activity and cellular homeostasis^31^. Conversely, abnormal depolarization of mitochondrial membrane potential not only impairs the mitochondrial capacity to generate reactive oxygen species and ATP but may also be intertwined with oxidative phosphorylation and glycolytic metabolic reprogramming, thereby inducing T-cell terminal exhaustion and compromising their antitumor efficacy^32^. This distinct functional trait of T cells is particularly pronounced in prostate cancer and is widely considered a significant factor contributing to resistance against immune therapy in this malignancy^33^. Furthermore, the metabolic hallmark of T-cell dysregulation and the transcriptional profile of exhaustion are viewed as key culprits promoting the progression of prostate cancer^34^, particularly in patients undergoing androgen receptor (AR) inhibitor treatment, as one of the potential functions of AR inhibitors is to reactivate the cytotoxic capacity of pre-exhausted T cells, yet this effect is invalid terminally exhausted T cells^35^, ^36^. This, in turn, suggests that a crucial pivot for overcoming the inefficacy of AR inhibitor combined with immunotherapy in prostate cancer lies in restoring the mitochondrial function of T cells.

Overall, our study has identified new pathways linking metabolic disorders and immune escape in prostate cancer, which is expected to provide key new targets for immunotherapy of prostate cancer.

## Supplementary Material

Supplementary materials and methods.

## Abbreviations

PGE2: prostaglandin E2
ABCC4: ATP-binding cassette protein C4
MRP4: Multidrug resistance protein 4
TILs: Tumor-infiltrating lymphocytes
AR: Androgen receptor
PRAD: Prostate adenocarcinoma
ANOVA: Analysis of Variance
KO: Knock out
KD: Knock down
OE: Over expression
EP2: Prostaglandin E receptor 2
EP4: Prostaglandin E receptor 4
OXPHS: Oxidative Phosphorylation
