# Supplementary material for: Tumor ABCC4-mediated release of PGE2 induces CD8+ T cell dysfunction and impairs PD-1 blockade in prostate cancer
Source: Int J Biol Sci. 2024 Aug 19;20(11):4424–37. doi: 10.7150/ijbs.99716 (PMC11380449; doi:10.7150/ijbs.99716)
Supplement: Supplementary file 1 — Supplementary materials and methods. [file ijbsv20p4424s1.pdf]

Tumor ABCC4-mediated Release of PGE2 induces CD8+ T cell Dysfunction and Impairs PD-1 Blockade in Prostate Cancer.

Supplementary methods

Quantitative PCR with reverse transcription

For RNA extraction, a Fastagen fast RNA extraction kit was used, and 1000 ng of RNA was used for complementary DNA synthesis with a cDNA synthesis kit from Takara. The cDNA was then diluted 1:10 for quantitative PCR (qPCR). The cDNAs were mixed with gene-specific primers listed in Supplementary Tables and SYBR green PCR Master Mix from Vazyme, and qRT-PCR was performed on an Applied Biosystems 8100HT Fast Real-Time PCR system. The values were normalized to the expression of the housekeeping gene β-actin. For qPCR of tumor cells derived from murine models in vivo, we separated the tumor mass, grinded physically, filtered with cell filter and then performed the gradient centrifugation using a density of 38% Percoll. After lysing red blood cells, we collected the mixture of immune cells and tumor cells, separated tumor cells (CD45 negative) after magnetic beads sorting and undertook qPCR analysis of these tumor cells.

CCK-8 assay

Renca cells after corresponding treatment in different groups were respectively seeded in 96-well culture plates (1×10^4/well) and incubated overnight at 37°C in a 5% CO2

incubator. The cells inoculated the day before were washed twice with PBS and then added with 100μl 1640 medium. Discard the old solution at 0, 24, 48 and 96 h after liquid exchange. Add 90μl medium and 10ul CCK-8 to each well of the 96-well test plate and incubate at 37°C for 2 h. A microplate reader (BioTek Instruments, Inc., Winooski, VT, USA) was used to measure OD values at 450nm for each experimental well and to detect changes in cell proliferation ability in each group.

Cell apoptosis assay

Apoptosis was analyzed using a flow cytometry assay (Becton Dickinson). For co-cultivation experiments, we first collected lymphocytes from Balb/c spleens, and then collected $CD8^+$ T cells purified by positive selection with magnetic beads from lymphocytes isolated from Balb/c mouse spleen and stimulated them with anti-CD3, anti-CD28 antibody and β-mercaptoethanol for 48 hours.

Cell Transfection

Vehicle plasmids V101-3flag and PCDH-H1 were gifts from the Laboratory of Biliary and Pancreatic Disease in Tongji hospital. Neofect was used to transfect the plasmids. Lentivirus-based short hairpin RNAs (shRNAs) were purchased from Sigma (USA). For RNA interference, gene-specific shRNA in combination with PSPAX and PMD2G were transfected into 293T cells. Twenty-four hours post transfection, the culture medium was replaced with fresh culture medium with 10% FBS. After 24 h, the medium without 293T cells was harvested, filtered and mixed with the medium containing cultured tumor cells. 3 days later, puromycin (10 μg mL^{-1}) was used to select positive renal cancer cells infected with shRNAs.
